# The Immunity Conferred by an Attack of Infectious Disease

**Published:** 1895-08

**Authors:** 


### THE IMMUNITY CONFERRED BY AN ATTACK OF INFECTIOUS DISEASE.

Maiselis has published important statistics bearing on the subject of the length of the periods of immunity conferred by attacks of infectious disease. This well-known principle, which is the basic principle of the work of Jenner, Pasteur, Koch, Behring, and all of the workers in the field of serum therapeutics, has been misstated by various writers on medicine, some of whom affirm that the survival from one attack of an infectious disease confers lifelong immunity. Gregory, for instance, says: "The immunity against a second attack of an infectious disease is one of the most universal and important principles in pathology. It is applicable not only to variola, measles, etc., but to all diseases which are due to a poisonous influence of miasm." Samuel says: "By the survival of a single attack of an infectious disease, immunity is generally conferred for the remainder of life." The same opinion has been expressed by Hebra, Henoch, Thomas, with regard to the acute exanthemata; by Griesinger, Murchinson, Zuelzer, as to typhoid fever; Audouard, as to Asiatic cholera.

On account of the great theoretical and practical interest of the question, Maiselis has collected from literature the following authenticated cases of repeated attacks of infectious diseases in the same patient:

*Smallpox.*—Two attacks, 526 cases; three attacks, 9; seven attacks, 1; total, 536.

*Scarlet Fever.*—Two attacks, 144 cases; three attacks, 7; four attacks, 1; eight attacks, 1; seventeen attacks, 1; total, 154.

*Measles.*—Two attacks, 103 cases; three attacks, 3; total, 106.

*Typhoid Fever.*—Two attacks, 203 cases; three attacks, 5; four attacks, 1; total, 209.

*Asiatic Cholera.*—Two attacks, 29 cases; three attacks, 3; four attacks, 2; total, 34.

In order to avoid the danger of including cases in which relapse might have been mistaken for a second attack, only those cases have been included in the preceding tables where the interval between the attacks was sufficiently long to preclude the possibility of the second or third attacks being relapses.

Considering the fact that all cases of second attacks of infectious disease are not recognized and that the deeply rooted belief in immunity among the laity, as well as among physicians, often renders the diagnosis of a second attack difficult to establish, one is led to believe that repeated attacks of infectious diseases may not belong to the rarities in medicine. This consideration also tends to establish the analogy between the immunity conferred by natural and artificial processes. The quantitative principle of immunity suggested by Ehrlich and systematically elaborated by Behring applies also to the natural processes of immunization.

It seems probable that so many difficulties would surround an exact determination of the period of immunity conferred by a given attack of infectious disease, that no exact statement of the safety period could be applied to particular cases. If a child is taken with scarlet fever, we have no means of estimating the amount of toxin absorbed, or the length of time the so-called natural antitoxin of the disease is present in effective quantity. The period of immunity conferred by the injection of given quantities of the artificial antitoxin of approximately known strength is a matter of doubt and study; and if the estimated length of the period of immunity is so difficult to compute in a case where we can approximately calculate the amount of antitoxin injected, how much more difficult will it be to calculate that conferred by the absorption of an unknown amount of toxin, and consequent antitoxin production?—*Boston Medical and Surgical Journal.*

